# Second-line therapy after *nab*-paclitaxel plus gemcitabine or after gemcitabine for patients with metastatic pancreatic cancer

**DOI:** 10.1038/bjc.2016.185

**Published:** 2016-06-28

**Authors:** E Gabriela Chiorean, Daniel D Von Hoff, Josep Tabernero, Robert El-Maraghi, Wen Wee Ma, Michele Reni, Marion Harris, Robert Whorf, Helen Liu, Jack Shiansong Li, Victoria Manax, Alfredo Romano, Brian Lu, David Goldstein

**Affiliations:** 1 Division Oncology, Department of Medicine, University of Washington, 825 Eastlake Avenue E, G4-833, Seattle, WA 98109-1023, USA; 2 Translational Genomics Research Institute and HonorHealth, 445 North Fifth Street, Suite 600, Phoenix, AZ 85004, USA; 3 Vall d'Hebron Institute of Oncology (VHIO), P Vall d'Hebron 119-129, Barcelona 08035, Spain; 4 Royal Victoria Hospital Barrie Canada, 201 Georgian Drive, Barrie, Ontario, Canada L4M 6M2; 5 Roswell Park Cancer Institute, 665 Elm Street, Buffalo, NY 14203, USA; 6 San Raffaele Scientific Institute, Via Olgetina 60, 20132 Milan, Italy; 7 Monash Health, 246 Clayton Road, Melbourne VIC 3168, Australia; 8 Florida Cancer Specialists, 2401 60th Street Ct W, Bradenton, FL 34209-5500, USA; 9 Celgene Corporation, 86 Morris Avenue, Summit, NJ 07901, USA; 10 Department of Medical Oncology, Prince of Wales Hospital, South Sydney Illawarra, Barker Street, Sydney NSW 2031, Australia

**Keywords:** metastatic pancreatic cancer, *nab*-paclitaxel, gemcitabine, MPACT, first line, second line

## Abstract

**Background:**

This exploratory analysis evaluated second-line (2L) therapy for metastatic pancreatic cancer in a large phase 3 trial (MPACT).

**Methods:**

Patients who received first-line (1L) *nab*-paclitaxel+gemcitabine (*nab*-P+Gem) or Gem were assessed for survival based on 2L treatment received. Multivariate analyses tested influence of treatment effect and prognostic factors on survival.

**Results:**

The majority of 2L treatments (267 out of 347, 77%) contained a fluoropyrimidine (5-fluorouracil or capecitabine). Median total survival (1L randomisation to death) for patients who received 2L treatment after 1L *nab*-P+Gem *vs* Gem alone was 12.8 *vs* 9.9 months (*P*=0.015). Median total survival for patients with a fluoropyrimidine-containing 2L therapy after *nab*-P+Gem *vs* Gem was 13.5 *vs* 9.5 months (*P*=0.012). Median 2L survival (duration from start of 2L therapy to death) was 5.3 *vs* 4.5 months for *nab*-P+Gem *vs* Gem, respectively (*P*=0.886). Factors significantly associated with longer post-1L survival by multivariate analyses included 1L *nab*-P+Gem, receiving 2L treatment, longer 1L progression-free survival, and Karnofsky performance status⩾70 and neutrophil-to-lymphocyte ratio⩽5 at the end of 1L treatment.

**Conclusions:**

These findings support the use of 2L therapy for patients with metastatic pancreatic cancer. Fluoropyrimidine-containing treatment after 1L *nab*-P+Gem is an active regimen with significant clinical effect.

Pancreatic cancer is known for its aggressiveness, which is evident from mortality rates nearly equal to incidence (World Health Organization, 2015). Current estimates rank pancreatic cancer as the fourth leading cause of cancer-related mortality in the United States (American Cancer Society, 2015); however, it is projected to become the second leading cause of cancer death by 2030 (Rahib *et al*, 2014). Unfortunately, ∼80% of patients are initially diagnosed with advanced disease (53% with metastatic disease and 28% with regional disease that has spread to lymph nodes (Surveillance, Epidemiology, and End Results Program, 2016)). Treatment plans for advanced pancreatic cancer over the past decade have consisted primarily of only one line of therapy (Abrams *et al*, 2014; Smyth *et al*, 2015). This may have been, at least in part, due to the aggressive nature of the disease and the lack of effective treatment options.

Recent results from two phase-3 trials (MPACT and PRODIGE) have demonstrated clinically meaningful, significantly longer overall survival (OS) with *nab*-paclitaxel+gemcitabine (*nab*-P+Gem) and FOLFIRINOX (folinic acid, 5-fluorouracil (5-FU), irinotecan, and oxaliplatin) regimens, respectively, *vs* Gem monotherapy, the standard for treating advanced pancreatic cancer since 1997 (Burris *et al*, 1997; Conroy *et al*, 2011; Von Hoff *et al*, 2013). The efficacy advantages observed for these new first-line (1L) therapies suggest greater opportunities for using second-line (2L) therapies in metastatic pancreatic cancer. The National Comprehensive Cancer Network currently recommends that if a Gem-based chemotherapy (such as with *nab*-P+Gem) is used as 1L therapy, the 2L therapy should be a fluoropyrimidine-based regimen, including combination therapies for fit patients (The National Comprehensive Cancer Network, 2015).

A growing number of clinical trials are now examining the efficacy of 2L therapy for metastatic pancreatic cancer, including the recent phase 3 NAPOLI-1 study, which led to approval of MM-398 in the 2L setting (Gill *et al*, 2014; Oettle *et al*, 2014; Wang-Gillam *et al*, 2015). As *nab*-P+Gem has become a standard option in the 1L setting, the identification of 2L therapies that will be effective after 1L *nab*-P+Gem is important. This *post*-*hoc* analysis was designed to evaluate survival outcomes associated with 2L treatment in patients enroled in the MPACT trial.

## Patients and methods

Patients received *nab*-P+Gem or Gem alone as 1L treatment for metastatic pancreatic cancer in the MPACT trial, as previously described (Von Hoff *et al*, 2013) Patients were treated until disease progression, unacceptable toxicity, or patient or physician decision. After treatment, OS and information on subsequent anticancer therapy were monitored on a monthly basis for 6 months and then every 3 months until death, study closure, or a period of 3 years had elapsed after treatment discontinuation, whichever occurred first. This evaluation was conducted by record review and/or telephone contact with the patient's treating physician. Data on subsequent therapies included only the type of treatment administered and the date that therapy was initiated. After the closure of the MPACT trial (NCT00844649) in 2013, an observational extension study was initiated to gather additional survival information on patients who were still alive (NCT02021500). The data collected from the extension study are included in this *post*-*hoc* evaluation.

Overall survival was analysed by the Kaplan–Meier method. Total OS was defined as time from 1L randomisation to death. For comparisons of patients who did *vs* did not receive 2L treatment, analyses were performed on post-1L OS, which was defined as the survival time from the end of 1L therapy to death. Second-line OS (OS2) was defined as the survival time from the start of 2L therapy to death.

Baseline characteristics and efficacy data for patients who received any 2L or no 2L therapy were derived from the treated population in the 1L setting. Three multivariate analyses were carried out to evaluate factors associated with (1) total OS, (2) post-1L OS (to understand the effect of 2L treatment), and (3) OS2 (only in patients who received 2L treatment). Each employed a Cox proportional hazards model to evaluate association of factors with OS. The multivariate analysis for total OS evaluated effects of 1L treatment, 2L treatment, and the following prognostic factors at baseline: geographic region (North America *vs* other), age, Karnofsky performance status (KPS, 70–80 *vs* 90–100), neutrophil-to-lymphocyte ratio (NLR, ⩽5 *vs*>5), pancreatic cancer primary location (head *vs* body/tail), stage at initial diagnosis, carbohydrate antigen 19-9 (CA19-9; evaluated as a continuous variable), presence of liver metastases, presence of peritoneal carcinomatosis, previous Whipple procedure, presence of biliary stent, presence of pulmonary metastases, and number of metastatic sites. The analysis for post-1L OS evaluated the effects of 1L treatment (*nab*-P+Gem *vs* Gem), 2L treatment (yes *vs* no), 1L progression-free survival (PFS; longer *vs* shorter than the median PFS of 4.4 months observed for the entire intention-to-treat (ITT) population), region (North America *vs* other), number of metastatic sites at baseline, and the following factors at the end of 1L treatment: age, KPS (70–80 *vs*⩽60 and 90–100 *vs*⩽60), and NLR (⩽5 *vs* >5). The analysis of OS2 evaluated effects of 1L treatment (*nab*-P+Gem *vs* Gem), 1L PFS (longer *vs* shorter than the median of 4.4 months observed for the entire ITT population), type of 2L treatment (combination *vs* monotherapy), region (North America *vs* other), number of metastatic sites at baseline, and the following factors at the end of 1L treatment: age, KPS (70–80 *vs* ⩽60 and 90–100 *vs* ⩽60), and NLR (⩽5 *vs*>5). For each analysis, a stepwise procedure was performed, to evaluate the treatment effect and identify the possible factors associated with OS. A significance level of 0.20 was required for entry into the model. A significance level of 0.10 was required to remain in the model.

Both MPACT and the observational extension of the MPACT study (NCT02021500) were conducted in accordance with the amended Declaration of Helsinki. The local institutional review boards or independent ethics committees approved the protocol and written informed consent was obtained from all patients.

## Results

### Patients and 2L treatments

Patient enrolment in MPACT occurred between May 2009 and April 2012 (ITT population: *n*=431 for *nab*-P+Gem and *n*=430 for Gem alone). A total of 823 of these 861 patients were treated: 421 with *nab*-P+Gem and 402 with Gem alone. After the MPACT trial closed, 45 surviving patients continued to be followed in the observational extension study (*n*=26 for *nab*-P+Gem and *n*=19 for Gem alone). The numbers of patients who received 2L treatment were similar between treatment arms (*n*=170 of 421 (40%) for *nab*-P+Gem and 177 of 402 (44%) for Gem alone). Most of the 2L regimens contained a fluoropyrimidine (5-FU or capecitabine): 132 of 170 (78%) in the *nab*-P+Gem arm and 135 of 177 (76%) in the Gem-alone arm; the majority of these were combination treatments (98 of 132 (74%) and 107 of 135 (79%), respectively), with the remainder being fluoropyrimidine monotherapy. In addition, 11 patients who received 1L Gem alone received *nab*-P monotherapy as 2L treatment (26% of the ‘other than fluoropyrimidine-containing' category) and 7 patients received *nab*-P+Gem. Of the 347 patients who received a 2L therapy, 69 (20%) received a third-line therapy (29 in the *nab*-P+Gem arm and 40 in the Gem-alone arm).

Baseline characteristics of patients who received 2L treatment were generally well balanced at the time of initial randomisation to 1L treatment (Table 1). However, compared with the ITT population, a higher percentage of patients who received 2L therapy had a KPS of 90–100 (68% for patients who received 1L *nab*-P+Gem and a 2L therapy, and 75% for patients who received 1L Gem alone and a 2L therapy *vs* 58% and 62%, respectively, in the ITT population). Baseline characteristics were also evaluated by specific 2L treatment received (Supplementary Table 1). Patient characteristics were relatively well balanced among the subgroups, except that patients in the *nab*-P+Gem and Gem-alone arms, who received 2L FOLFIRINOX (*n*=18 and 17, respectively), when compared with those in the ITT arms, were younger (median ages, 53.5 and 56.0 *vs* 62.0 and 63.0 years), fitter (KPS 90–100, 72% and 76% *vs* 58% and 62%), and less likely to have a higher tumour burden (>3 metastatic sites; 0 and 12% *vs* 14% and 15%); however, baseline CA19-9 was similar or higher in patients who received 2L FOLFIRINOX *vs* the ITT population (median 5539 and 2368 U ml^−1^ *vs* 2294 and 2759 U ml^−1^).

As MPACT was designed as a prospective 1L study, no information was collected on patient characteristics at the start of 2L therapy. However, as a surrogate, a few key patient characteristics at the end of 1L treatment, such as KPS, CA19-9, and NLR, are reported in Table 2, to help understand some patient characteristics of those who received a subsequent therapy.

Among patients who received 2L therapy, most discontinued 1L treatment due to disease progression (59% and 74% in the *nab*-P+Gem and Gem-alone arms, respectively). Fewer patients (26% and 14% of patients in the *nab*-P+Gem and Gem-alone arms, respectively) discontinued 1L treatment due to adverse events. The median durations of 1L therapy in patients who received 2L therapy were 5.3 months for *nab*-P+Gem and 3.7 months for Gem alone (Supplementary Figure S1).

### Efficacy in the 1L setting

**Figure 1:**
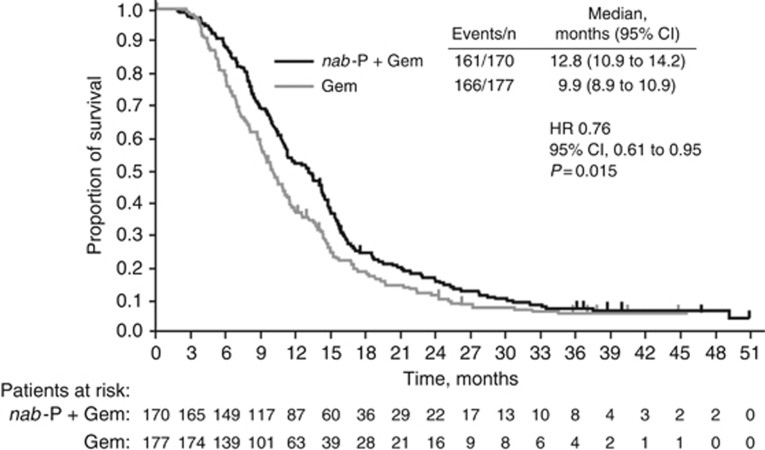
**Total OS in patients who received 2L therapy.** Gem=gemcitabine; HR=hazard ratio; *nab*-P=*nab*-paclitaxel.

**Figure 2:**
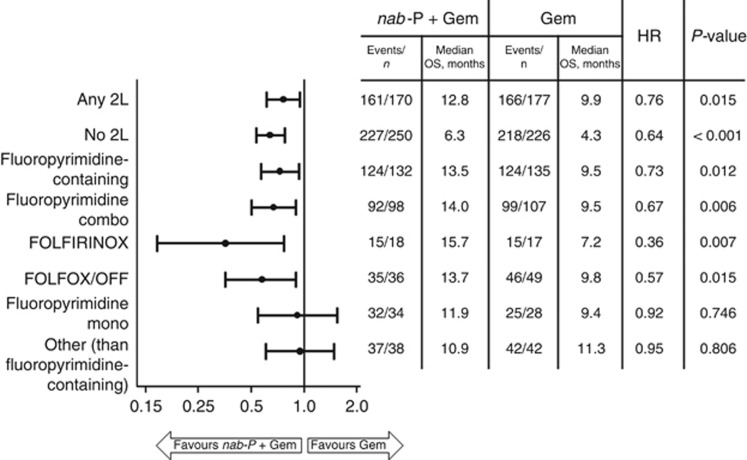
**Forest plot of total OS in subgroups defined by 2L therapy.** FOLFIRINOX=folinic acid, 5-fluorouracil, irinotecan, and oxaliplatin; FOLFOX=folinic acid, 5-FU, and oxaliplatin; Gem=gemcitabine; HR=hazard ratio; mono=monotherapy; *nab*-P=*nab*-paclitaxel; OFF=oxaliplatin, folinic acid, and 5-FU.

Pooling both 1L treatment arms, the median total OS from 1L randomisation for patients who did (*n*=347) and did not (*n*=476) receive a 2L therapy was 10.9 and 5.4 months, respectively. In the cohort of patients who received any 2L therapy, efficacy was greater with *nab*-P+Gem *vs* Gem alone in terms of total OS from 1L randomisation (median=12.8 *vs* 9.9 months, HR=0.76, 95% CI=0.61–0.95, *P*=0.015; Figures 1 and 2) and overall response rate during 1L therapy (28% *vs* 9%, *P*<0.001). First-line PFS was longer for patients who received 1L *nab*-P+Gem *vs* Gem alone, although the difference was not statistically significant (median=7.6 *vs* 5.4 months, HR=0.75, 95% CI=0.55–1.02, *P*=0.067; Supplementary Table 2). For patients who did not receive 2L therapy, median OS was 6.3 *vs* 4.3 months (HR=0.64, 95% CI=0.53–0.78, *P*<0.001) and overall response rate during 1L treatment was 21% *vs* 7% (*P*<0.001), respectively.

In the cohort of patients who received 2L therapy, a lower percentage of patients in the *nab*-P+Gem *vs* Gem-alone arm experienced progressive disease before week 8 (9% *vs* 15%, respectively). In the cohort of patients who did not receive 2L therapy, 6% of those who received 1L *nab*-P+Gem *vs* 12% of those who received Gem alone had progressive disease before week 8. To further eliminate potential bias of poor prognostic factors in the group of patients who did not receive 2L therapy, total OS was examined in a pooled-treatment-arm cohort of only those patients with KPS 90–100 and NLR ⩽5 at the end of 1L treatment. Overall survival was longer for those who did (*n*=116) *vs* did not (*n*=70) receive 2L therapy: median OS 14.1 *vs* 9.6 months (HR=0.72, 95% CI=0.52–0.99, *P*=0.042).

Baseline factors found to significantly associate with longer total OS in a multivariate analysis included 1L treatment with *nab*-P+Gem, use of any 2L therapy, better KPS at baseline (90–100 *vs* 70–80), no liver metastases at baseline, NLR at baseline ⩽5 *vs* >5 (*P*<0.001 for each), and lower CA19-9 at baseline (evaluated as a continuous variable, *P*=0.005; Table 3).

The total OS in the group of patients who received a fluoropyrimidine-containing regimen was significantly longer for those who received 1L *nab*-P+Gem *vs* Gem alone (median=13.5 *vs* 9.5 months, HR=0.73, 95% CI=0.57–0.93, *P*=0.012; Figure 2). The longest median total OS values in the *nab*-P+Gem arm were for patients who received fluoropyrimidine-containing combinations, such as FOLFIRINOX (*n*=18, median=15.7 months) and FOLFOX (folinic acid, 5-FU, and oxaliplatin; *n*=36, median=13.7 months); 2 patients who received 2L FOLFIRI (folinic acid, 5-FU, and irinotecan) after *nab*-P+Gem had a total OS of 5.8 and 8.9 months.

A minority of patients (23%) received 2L therapies other than fluoropyrimidine-containing regimens; these included erlotinib and various investigational agents. In addition, some patients in the 1L Gem-alone arm received a *nab*-P-based regimen for 2L treatment. Median total OS for patients who received 1L Gem alone and 2L *nab*-P monotherapy (*n*=11) or *nab*-P+Gem (*n*=7) was 8.8 and 7.9 months, respectively.

### Post-1L survival (from end of 1L therapy to death)

To understand the differences between outcomes in patients who did *vs* did not receive a 2L therapy, the duration of survival from the end of 1L therapy was examined. A multivariate analysis including treated patients in MPACT identified the following factors significantly associated with longer post-1L survival: 1L treatment with *nab*-P+Gem, use of any 2L therapy, NLR⩽5 *vs*>5 at end of 1L therapy, better KPS at end of 1L therapy (90–100 *vs* ⩽60 and 70–80 *vs* ⩽60; *P*<0.001 for each), and 1L PFS ⩾4.4 months *vs*<4.4 months (the median for the ITT population, *P*=0.002; Table 3).

### OS2: survival time from the start of 2L therapy to death

**Figure 3:**
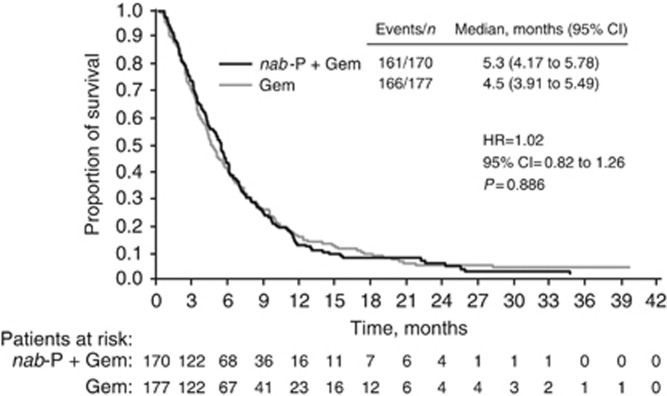
**Second-line OS (OS2), defined as survival time from the start of 2L therapy to death, in patients who received 2L therapy.** Gem=gemcitabine; HR=hazard ratio; *nab*-P=*nab*-paclitaxel.

For patients who received any 2L therapy (*n*=347), OS2 was similar for patients who received 1L *nab*-P+Gem *vs* Gem alone (median=5.3 months for *nab*-P+Gem *vs* 4.5 months for Gem alone; all 2L treatments; HR for OS=1.02, 95% CI=0.82–1.26, *P*=0.886; Figure 3 and Supplementary Table 3). Among patients who received a fluoropyrimidine-containing 2L treatment, median OS2 was 5.7 months for *nab*-P+Gem *vs* 4.5 months for Gem alone (HR for OS=0.94, 95% CI=0.73–1.20, *P*=0.606; Supplementary Table 3).

Among patients who received any 2L therapy, factors found to significantly associate with longer OS2 in a multivariate analysis included fewer metastatic sites at baseline, NLR ⩽5 at end of 1L treatment, and KPS 90–100 *vs* ⩽60 at end of 1L treatment (*P*<0.05 for each; Table 3).

In general, groups with longer median 1L treatment duration demonstrated a trend towards longer median OS2 (Supplementary Figure 1).

## Discussion

The overall results of this analysis demonstrated that 2L treatment was feasible and appeared to confer an OS benefit compared with no 2L treatment in the large, randomised MPACT patient population. Indeed, the median OS of patients in the *nab*-P+Gem group who received a 2L treatment was over 4 months longer than the median OS with *nab*-P+Gem in the ITT population (12.8 *vs* 8.7 months; Von Hoff *et al*, 2013) and over 6 months longer than the median OS with *nab*-P+Gem in patients who did not receive 2L therapy (12.8 *vs* 6.2 months). A similar effect was observed in the Gem-alone arm (median OS, 9.9 for those who received 1L Gem alone and a 2L treatment *vs* 6.6 months in the ITT population). A matched cohort analysis of patients with good prognostic factors (KPS 90–100 and NLR ⩽5) at the end of 1L treatment supported a benefit for 2L treatment, as did multivariate analyses of total OS and post-1L survival, further confirming that subsequent therapy is beneficial. Patients who received *nab*-P+Gem *vs* Gem alone for 1L therapy followed by fluoropyrimidine combination therapy as the 2L treatment achieved a median OS2 of 6.0 *vs* 4.6 months (HR=0.87, 95% CI= 0.66–1.16, *P*=0.352), consistent with the idea that more effective 1L therapy may have an important role in achieving optimal benefit from specific subsequent therapies. In addition, these data suggest that fluoropyrimidine combination regimens are feasible as 2L therapy after *nab*-P+Gem and confer better results than monotherapy after 1L combination therapy.

The median durations of OS2 (all 2L therapies combined, 5.3 months for *nab*-P+Gem and 4.5 months for Gem alone) were comparable to those reported in the PRODIGE trial of FOLFIRINOX *vs* Gem as 1L therapy (4.4 months in both arms; Conroy *et al*, 2011). Combination 2L therapies in MPACT appeared to result in longer survival. Some factors that might explain this effect are the efficacy of the specific 2L treatment and the underlying patient biology that allowed patients to receive that therapy. For example, FOLFIRINOX after *nab*-P+Gem resulted in the longest OS of all treatments analysed (this result must be interpreted with caution given the small sample size (*n*=18)); however, FOLFIRINOX was given to patients with favourable characteristics at the end of 1L therapy (e.g., those with better performance status), making it difficult to determine how much of the longer survival was due to the efficacy of FOLFIRINOX *vs* inherent patient factors. Indeed, separate multivariate analyses demonstrated significant associations of total OS and post-1L OS with 1L *nab*-P+Gem *vs* Gem alone, receipt of 2L therapy, and KPS and NLR (at baseline for total OS and at the end of 1L therapy for post-1L OS). A third multivariate analysis identified baseline number of metastatic sites, as well as NLR and KPS at the end of 1L treatment, as significantly associated with OS2. These clinical characteristics at the end of 1L therapy will help clinicians identify which patients will achieve the greatest benefit from 2L therapy. More research to distinguish the extent of benefit of treatment as opposed to favourable prognostic biology is required.

Sequential treatment planning for metastatic pancreatic cancer is a new concept made possible by therapeutic advances such as *nab*-P+Gem and FOLFIRINOX. Disease management previously focused on palliation of symptoms after progression following initial therapy. However, the results from this study and others (Supplementary Table 4) suggest that care plans for patients with metastatic pancreatic cancer are evolving to include multiple lines of therapy (Conroy *et al*, 2011; Gill *et al*, 2014; Oettle *et al*, 2014; Portal *et al*, 2015; Wang-Gillam *et al*, 2015; Kobayashi *et al*, 2016; Schmidt *et al*, 2016). In addition, although little is known about interactions between specific 1L and 2L therapies in terms of overall efficacy, groups with longer median duration of 1L therapy generally had longer median OS2 in this subanalysis (Supplementary Figure 1) and a multivariate analysis demonstrated that PFS duration in the 1L setting was associated with post-1L survival. The association between 1L PFS and post-1L survival is consistent with a previous study (Reni *et al*, 2008). Taken together, these findings highlight the benefit that a patient could theoretically derive from maximizing duration of 1L therapy (in the absence of disease progression) through careful monitoring of patient tolerability and enacting dose modifications when necessary.

Genomic analyses may hold promise in guiding treatment selection based on tumour biology (Sausen *et al*, 2015) and, although there are often valid reasons for a patient not to be offered 2L therapy (e.g., toxicity and patient decisions), a broader assortment of options and information on potential benefit of specific treatment sequences will undoubtedly improve patient care.

Interpretation of these results is subject to a number of limitations. First, as a *post*-*hoc* analysis of a prospective 1L study, no measures were enacted to ensure controlled comparisons among groups with regard to 2L therapy. Second, beyond the start date and description of components, details on 2L regimens were not collected, resulting in a lack of information on doses, schedules, duration of 2L therapy, disease progression, and tolerability of 2L treatment. In addition, patient characteristics were not specifically recorded at the initiation of 2L therapy, although certain key factors, including age, CA19-9, and NLR were collected at the end of 1L therapy. Third, when considering the longer OS in patients who received 2L treatment *vs* the ITT population, it is important to note that this cohort, by definition, excluded patients who died too early to receive 2L treatment. Fourth, there are no data from this analysis on patients who received 2L liposomal irinotecan, which recently became the only approved 2L option for metastatic pancreatic cancer (Merrimack Pharmaceuticals, 2015), nor was irinotecan a highly used option (e.g., as a component of FOLFIRI) at the time of trial conduct. Most patients in the MPACT study received oxaliplatin- and fluoropyrimidine-containing combination treatments (e.g., FOLFOX or OFF (oxaliplatin, folinic acid, and 5-FU)), per established treatment recommendations at the time the study was conducted.

The findings from this analysis support the increasing use of 2L therapy in patients with metastatic pancreatic cancer. Specifically, these results showed that a fluoropyrimidine-containing treatment after 1L *nab*-P+Gem or Gem alone may be an active treatment sequence with favourable clinical benefit. Further guidance for the use of 2L therapy will depend on enhanced identification of biologic predictors of 2L treatment benefit, development of more active regimens, and a better understanding of how an effective 1L therapy may influence clinical benefit in subsequent treatments.

## Figures and Tables

**Table 1:** Baseline characteristics at time of 1L randomisation

	**ITT**		**Any 2L treatment**		**No 2L treatment**	
	***nab*****-P+Gem**	**Gem**	***nab*****-P+Gem**	**Gem**	***nab*****-P+Gem**	**Gem**
*n*	431	430	170^a^	177^a^	250^a^	226^a^
Age, median, years	62.0	63.0	61.0	62.0	63.0	64.0
KPS, %						
90–100	58	62	68	75	52	51^b^
70–80	42	38	32	25	48	49^b^
CA19-9	*n*=379	*n*=371	*n*=152	*n*=162	*n*=225	*n*=206
U ml^−1^, median	2294	2759	2644	2096	1951	3733
⩾59 × ULN, %	52	53	49	46	45	50
Region, %						
North America	62	63	66	63	59	60
Other	38	38	34	37	41	40
No. metastatic sites, %						
1–3	86	85	88	88	85	84
>3	14	15	12	12	15	16
NLR, %	*n*=426	*n*=426				
⩽5	62	65	75	73	55	58
>5	38	35	25	27	45	42

Abbreviations: 1L=first-line; 2L=second-line; CA19-9=carbohydrate antigen 19-9; Gem=gemcitabine; ITT=intention-to-treat; KPS=Karnofsky performance status; *nab*-P=*nab*-paclitaxel; NLR=neutrophil-to-lymphocyte ratio; ULN=upper limit of normal.

a Only patients who received 1L treatment (not the entire ITT population) were included in the Any 2L or No 2L treatment cohorts.

b Based on 225 evaluable patients.

**Table 2:** Patient characteristics at end of 1L treatment by 2L therapy received

	**Any 2L treatment**		**No 2L treatment**		**Fluoro-pyrimidine-containing**		**Fluoro-pyrimidine combo**		**FOLFIRINOX**		**FOLFOX/OFF**		**Fluoro-pyrimidine mono**		**Other (than fluoro-pyrimidine-containing)**^a^	
	***nab*****-P+Gem**	**Gem**	***nab*****-P+Gem**	**Gem**	***nab*****-P+Gem**	**Gem**	***nab*****-P+Gem**	**Gem**	***nab*****-P+Gem**	**Gem**	***nab*****-P+Gem**	**Gem**	***nab*****-P+Gem**	**Gem**	***nab*****-P+Gem**	**Gem**
*n*	170	177	250	226	132	135	98	107	18	17	36	49	34	28	38	42
Age, median, years	61.8	62.5	63.6	65.2	60.4	62.5	59.8	62.5	54.4	56.2	59.5	64.7	62.5	61.3	65.9	62.2
KPS																
*n*	170	176	238	219	132	135	98	107	18	17	36	49	34	28	38	41
90–100, %	43	48	26	22	43	45	47	48	50	59	44	35	32	36	42	56
80, %	37	34	28	31	38	35	38	35	39	12	31	51	38	36	34	32
70, %	11	10	19	19	8	12	6	11	6	29	6	10	15	14	21	5
⩽60, %	9	8	27	28	11	8	9	7	6	0	19	4	15	14	3	7
CA19-9																
* n*	144	131	168	120	112	99	84	76	16	10	32	33	28	23	32	32
U ml^−1^, median	276	380	246	1674	276	514	241	547	195	401	398	261	289	148	277	302
⩾59 × ULN, %	26	32	29	45	28	33	27	33	19	20	28	30	29	35	19	28
NLR ⩽5, %	74	67	59	44	77	64	77	65	72	71	69	63	79	61	61	76

Abbreviations: 1L=first-line; 2L=second-line; CA19-9=carbohydrate antigen 19-9; FOLFIRINOX=folinic acid, 5-fluorouracil, irinotecan, and oxaliplatin; FOLFOX=folinic acid, 5-fluorouracil, and oxaliplatin; Gem=gemcitabine; KPS=Karnofsky performance status; *nab*-P=*nab*-paclitaxel; NLR=neutrophil-to-lymphocyte ratio; OFF=oxaliplatin, folinic acid, and 5-fluorouracil; ULN=upper limit of normal.

a Regimens in the Other category included the following: *nab*-P+Gem (for the 1L Gem arm only), *nab*-P monotherapy (for the 1L Gem arm only), erlotinib-containing regimens, and other Gem-based combinations.

**Table 3:** Multivariate analyses of total OS, post-1L OS, and survival time from the start of 2L therapy to death (OS2)

**Covariate**^a^	**HR (95% CI)**	***P*****-value**
Total OS (includes treated patients with or without a 2L therapy; *n*=741)		
Treatment group (*nab*-P+Gem *vs* Gem alone)	0.63 (0.54–0.74)	<0.001
2L therapy (with *vs* without)	0.50 (0.43–0.59)	<0.001
NLR at baseline (⩽5 *vs* >5)	0.59 (0.52–0.70)	<0.001
KPS at baseline (70–80 *vs* 90–100)	1.33 (1.13–1.55)	<0.001
Presence of liver metastasis (yes *vs* no)	1.50 (1.21–1.85)	<0.001
CA19-9 level at baseline (continuous)	1.16 (1.05–1.29)	0.005
Age at baseline (<65 *vs* ⩾65 years)	0.88 (0.75–1.02)	0.089
Post-1L OS (includes treated patients with or without a 2L therapy; *n*=793)		
Treatment group (*nab*-P+Gem *vs* Gem alone)	0.73 (0.63–0.85)	<0.001
2L therapy (with *vs* without)	0.47 (0.40–0.54)	<0.001
NLR at end of 1L (⩽5 *vs* >5)	0.60 (0.52–0.70)	<0.001
**KPS at end of 1L**		
90–100 *vs* ⩽60	0.46 (0.37–0.57)	<0.001
70–80 *vs* ⩽60	0.57 (0.47–0.70)	<0.001
PFS, months (⩾4.4 *vs* <4.4)^b^	0.78 (0.67–0.91)	0.002
Geographic region (North America *vs* others)	0.86 (0.74–1.00)	0.051
OS2 (only patients who received 2L therapy; *n*=346)		
Number of metastatic sites	1.15 (1.02–1.29)	0.018
NLR at end of 1L (⩽5 *vs* >5)	0.76 (0.60–0.97)	0.027
**KPS at end of 1L**		
90–100 *vs* ⩽60	0.53 (0.35–0.81)	0.003
70–80 *vs* ⩽60	0.66 (0.44–1.00)	0.052

Abbreviations: 1L=first-line; 2L=second-line; CA19-9=carbohydrate antigen 19-9; CI=confidence interval; Gem=gemcitabine; HR=hazard ratio; KPS=Karnofsky performance status; *nab*-P=*nab*-paclitaxel; NLR=neutrophil-to-lymphocyte ratio; OS=overall survival; PFS=progression-free survival.

a Covariates tested for each multivariate analysis are listed in the Patients and Methods section.

b In this study, the median PFS for the entire intention-to-treat population was 4.4 months.

## References

[bib1] Abrams TA, Meyer G, Moloney J, Meyerhardt JA, Wolpin BW, Schrag D, Fuchs CS (2014) Patterns of chemotherapy (CT) use in a population-based US-wide cohort of patients (pts) with metastatic pancreatic cancer (MPC). J Clin Oncol 32(suppl 5): abstract 4131.

[bib2] American Cancer Society (2015) Cancer Facts and Figures 2015. American Cancer Society: Atlanta, GA, USA.

[bib3] Burris HA 3rd, Moore MJ, Andersen J, Green MR, Rothenberg ML, Modiano MR, Cripps MC, Portenoy RK, Storniolo AM, Tarassoff P, Nelson R, Dorr FA, Stephens CD, Von Hoff DD (1997) Improvements in survival and clinical benefit with gemcitabine as first-line therapy for patients with advanced pancreas cancer: a randomized trial. J Clin Oncol 15: 2403–2413. 9196156 10.1200/JCO.1997.15.6.2403

[bib4] Conroy T, Desseigne F, Ychou M, Bouche O, Guimbaud R, Becouarn Y, Adenis A, Raoul JL, Gourgou-Bourgade S, de la Fouchardiere C, Bennouna J, Bachet JB, Khemissa-Akouz F, Pere-Verge D, Delbaldo C, Assenat E, Chauffert B, Michel P, Montoto-Grillot C, Ducreux M (2011) FOLFIRINOX versus gemcitabine for metastatic pancreatic cancer. N Engl J Med 364: 1817–1825. 21561347 10.1056/NEJMoa1011923

[bib5] Gill S, Ko Y, Cripps C, Beaudoin A, Dhesy-Thind SK, Zulfiqar M, Salweski P, Do T, Cano PO, Lam W, Dowden SD, Grassin H, Stewart J, Moore MJ (2014) PANCREOX: a randomized phase 3 study of 5FU/LV with or without oxaliplatin for second-line advanced pancreatic cancer (APC) in patients (pts) who have received gemcitabine (GEM)-based chemotherapy (CT). J Clin Oncol 32(suppl 5): abstract 4022. 10.1200/JCO.2016.68.5776 27621395

[bib6] Kobayashi N, Tokuhisa M, Goto A, Endo I, Ichikawa Y (2016) Second-line chemotherapy by FOLFIRINOX with unresectable pancreatic cancer (phase I, II study). J Clin Oncol 34(suppl 4S): abstract 297.

[bib7] Merrimack Pharmaceuticals (2015) Onivyde (irinotecan liposome injection) [package insert], Cambridge, MA, USA.

[bib8] Oettle H, Riess H, Stieler JM, Heil G, Schwaner I, Seraphin J, Gorner M, Molle M, Greten TF, Lakner V, Bischoff S, Sinn M, Dorken B, Pelzer U (2014) Second-line oxaliplatin, folinic acid, and fluorouracil versus folinic acid and fluorouracil alone for gemcitabine-refractory pancreatic cancer: outcomes from the CONKO-003 trial. J Clin Oncol 32: 2423–2429. 24982456 10.1200/JCO.2013.53.6995

[bib9] Portal A, Pernot S, Tougeron D, Arbaud C, Bidault AT, de la Fouchardiere C, Hammel P, Lecomte T, Dreanic J, Coriat R, Bachet JB, Dubreuil O, Marthey L, Dahan L, Tchoundjeu B, Locher C, Lepere C, Bonnetain F, Taieb J (2015) Nab-paclitaxel plus gemcitabine for metastatic pancreatic adenocarcinoma after FOLFIRINOX failure: an AGEO prospective multicentre cohort. Br J Cancer 113: 989–995. 26372701 10.1038/bjc.2015.328 PMC4651133

[bib10] Rahib L, Smith BD, Aizenberg R, Rosenzweig AB, Fleshman JM, Matrisian LM (2014) Projecting cancer incidence and deaths to 2030: the unexpected burden of thyroid, liver, and pancreas cancers in the United States. Cancer Res 74: 2913–2921. 24840647 10.1158/0008-5472.CAN-14-0155

[bib11] Reni M, Berardi R, Mambrini A, Pasetto L, Cereda S, Ferrari VD, Cascinu S, Cantore M, Mazza E, Grisanti S (2008) A multi-centre retrospective review of second-line therapy in advanced pancreatic adenocarcinoma. Cancer Chemother Pharmacol 62: 673–678. 18172650 10.1007/s00280-007-0653-y

[bib12] Sausen M, Phallen J, Adleff V, Jones S, Leary RJ, Barrett MT, Anagnostou V, Parpart-Li S, Murphy D, Kay Li Q, Hruban CA, Scharpf R, White JR, O'Dwyer PJ, Allen PJ, Eshleman JR, Thompson CB, Klimstra DS, Linehan DC, Maitra A, Hruban RH, Diaz LA Jr, Von Hoff DD, Johansen JS, Drebin JA, Velculescu VE (2015) Clinical implications of genomic alterations in the tumour and circulation of pancreatic cancer patients. Nat Commun 6: 7686. 26154128 10.1038/ncomms8686 PMC4634573

[bib13] Schmidt SL, Durkal V, Jayavalsan SP, Thomas JP, Ritch PS, Erickson B, Christians KK, Tsai S, Evans DB, George B (2016) Outcomes in metastatic pancreatic adenocarcinoma (MPAC) patients treated with FOLFIRINOX (FFX)/FOLFOX(FX) and gemcitabine+nab-paclitaxel (NabG). J Clin Oncol 34(suppl 4S): abstract 397.

[bib14] Smyth EN, Bapat B, Ball DE, Andre T, Kaye JA (2015) Metastatic pancreatic adenocarcinoma treatment patterns, health care resource use, and outcomes in France and the United Kingdom between 2009 and 2012: a retrospective study. Clin Ther 37: 1301–1316. 25907619 10.1016/j.clinthera.2015.03.016

[bib15] Surveillance, Epidemiology, and End Results Program (2016) SEER Stat Facts Sheets: Pancreas Cancer, Bethesda, MD, USA.

[bib16] The National Comprehensive Cancer Network (2015) NCCN Clinical Practice Guidelines in Oncology: Pancreatic Adenocarcinoma, V2.2015, Washington, PA, USA.

[bib17] Von Hoff DD, Ervin T, Arena FP, Chiorean EG, Infante J, Moore M, Seay T, Tjulandin SA, Ma WW, Saleh MN, Harris M, Reni M, Dowden S, Laheru D, Bahary N, Ramanathan RK, Tabernero J, Hidalgo M, Goldstein D, Van Cutsem E, Wei X, Iglesias J, Renschler MF (2013) Increased survival in pancreatic cancer with nab-paclitaxel plus gemcitabine. N Engl J Med 369: 1691–1703. 24131140 10.1056/NEJMoa1304369 PMC4631139

[bib18] Wang-Gillam A, Li CP, Bodoky G, Dean A, Shan YS, Jameson G, Macarulla T, Lee KH, Cunningham D, Blanc JF, Hubner RA, Chiu CF, Schwartsmann G, Siveke JT, Braiteh F, Moyo V, Belanger B, Dhindsa N, Bayever E, Von Hoff DD, Chen LT NAPOLI-1 Study Group (2015) Nanoliposomal irinotecan with fluorouracil and folinic acid in metastatic pancreatic cancer after previous gemcitabine-based therapy (NAPOLI-1): a global, randomised, open-label, phase 3 trial. Lancet 387(10018): 545–557. 26615328 10.1016/S0140-6736(15)00986-1

[bib19] World Health Organization (2015) GLOBOCAN 2012: Estimated Cancer Incidence, Mortality, and Prevalence Worldwide in 2012, Lyon, France (accessed 17 September 2015).

