# Supplementary material for: Second-line therapy after nab-paclitaxel plus gemcitabine or after gemcitabine for patients with metastatic pancreatic cancer
Source: Br J Cancer. 2016 Jun 28;115(2):188–94. doi: 10.1038/bjc.2016.185 (PMC4947701; doi:10.1038/bjc.2016.185)
Supplement: Supplementary Table 1 [file bjc2016185x1.docx]

Supplemental Table 1. Baseline characteristics at time of first-line (1L) randomization in second-line (2L) therapy subgroups

|  | Fluoropyrimidine-containing | | Fluoropyrimidine combo | | FOLFIRINOX | | FOLFOX/OFF | | Fluoropyrimidine monotherapy | | Other (than fluoropyrimidine-containing) | |
| --- | --- | --- | --- | --- | --- | --- | --- | --- | --- | --- | --- | --- |
|  | *nab*-P + Gem | Gem | *nab*-P + Gem | Gem | *nab*-P + Gem | Gem | *nab*-P + Gem | Gem | *nab*-P + Gem | Gem | *nab*-P + Gem | Gem |
| n | 132 | 135 | 98 | 107 | 18 | 17 | 36 | 49 | 34 | 28 | 38 | 42 |
| Age, median, y | 59.5 | 62.0 | 59.0 | 62.0 | 53.5 | 56.0 | 58.5 | 64.0 | 61.5 | 60.0 | 65.0 | 61.5 |
| KPS, %  90-100  70-80 | 64  36 | 76  24 | 67  33 | 76  24 | 72  28 | 76  24 | 53  47 | 67  33 | 53  47 | 79  21 | 84  16 | 71  29 |
| CA19-9  U/mL, median ≥59×ULN, % | n=120  2637  51 | n=125  2172  47 | n=90  2601  51 | n=100  2206  48 | n=16  5539  56 | n=17  2368  53 | n=33  2650  53 | n=43  2005  43 | n=30  3522  50 | n=25  822  43 | n=32  2857  45 | n=37  1500  43 |
| Region, %  N America  Other | 64  36 | 66  34 | 72  28 | 73  27 | 89  11 | 82  18 | 78  22 | 80  20 | 41  59 | 39  61 | 71  29 | 55  45 |
| No. metastatic sites, %  1-3  >3 | 88  12 | 89  11 | 91  9 | 93  7 | 100  0 | 88  12 | 86  14 | 92  8 | 79  21 | 75  25 | 87  13 | 83  17 |
| NLR, %  ≤5  >5 | 74  26 | 72  28 | 75  25 | 70  30 | 72  28 | 71  29 | 78  22 | 69  31 | 72  28 | 81  19 | 76  24 | 74  26 |
|  | | | | | | | | | | | | |

CA19-9, carbohydrate antigen 19-9; FOLFIRINOX, folinic acid, 5-fluorouracil, irinotecan, and oxaliplatin; FOLFOX, folinic acid, 5-fluorouracil, and oxaliplatin; Gem, gemcitabine; KPS, Karnofsky performance status; *nab*-P, *nab*-paclitaxel; NLR, neutrophil-to-lymphocyte ratio; OFF, oxaliplatin, folinic acid, and 5-fluorouracil; ULN, upper limit of normal.
