# Supplementary material for: Second-line therapy after nab-paclitaxel plus gemcitabine or after gemcitabine for patients with metastatic pancreatic cancer
Source: Br J Cancer. 2016 Jun 28;115(2):188–94. doi: 10.1038/bjc.2016.185 (PMC4947701; doi:10.1038/bjc.2016.185)
Supplement: Supplementary Table 2 [file bjc2016185x2.docx]

Supplemental Table 2. First-line progression-free survival (PFS) in patients who received any second-line (2L) treatment

| PFS per 2L regimen | ***nab*-P + Gem** | **Gem** | **HR (95% CI)**  ***P* value** |
| --- | --- | --- | --- |
| Any 2L treatment, n (%)  **Median, mo (95% CI)** | 170/421 (40)  **7.3 (5.95 to 8.90)** | 177/402 (44)  **5.4 (4.07 to 5.72)** | **0.75 (0.548 to 1.023)**  **0.067** |
| Fluoropyrimidine-containing, n (%)  **Median, mo (95% CI)** | 132/170 (78)  **7.3 (5.62 to 8.87)** | 135/177 (76)  **5.4 (3.75 to 5.72)** | **0.79 (0.555 to 1.118)**  **0.179** |
| Fluoropyrimidine combo, n (%)  **Median, mo (95% CI)** | 98/132 (74)  **7.4 (5.52 to 8.87)** | 107/135 (79)  **5.4 (3.84 to 5.59)** | **0.71 (0.474 to 1.075)**  **0.103** |
| FOLFIRINOX, n (%)  **Median, mo (95% CI)** | 18/132 (14)  **7.5 (5.55 to 10.48)** | 17/135 (13)  **5.4 (3.75 to 5.59)** | **0.26 (0.086 to 0.784)**  **0.011** |
| FOLFOX/OFF, n (%)  **Median, mo (95% CI)** | 36/132 (27)  **7.3 (4.40 to 13.01)** | 49/135 (36)  **5.4 (3.71 to 7.43)** | **0.65 (0.326 to 1.289)**  **0.212** |
| Fluoropyrimidine mono, n (%)  **Median, mo (95% CI)** | 34/132 (26)  **6.6 (5.55 to 9.56)** | 28/135 (21)  **5.6 (3.52 to —)** | **0.92 (0.455 to 1.870)**  **0.821** |
| Other (than fluoropyrimidine-containing), n (%)  **Median, mo (95% CI)** | 38/170 (22)  **9.1 (5.39 to 13.11)** | 42/177 (24)  **5.5 (3.71 to 20.11)** | **0.58 (0.285 to 1.169)**  **0.121** |

FOLFIRINOX, folinic acid, 5-fluorouracil, irinotecan, and oxaliplatin; FOLFOX, folinic acid, 5-fluorouracil, and oxaliplatin; Gem, gemcitabine; HR, hazard ratio; mono, monotherapy; *nab*-P, *nab*-paclitaxel; OFF, oxaliplatin, folinic acid, and 5-fluorouracil.
