# Supplementary material for: Second-line therapy after nab-paclitaxel plus gemcitabine or after gemcitabine for patients with metastatic pancreatic cancer
Source: Br J Cancer. 2016 Jun 28;115(2):188–94. doi: 10.1038/bjc.2016.185 (PMC4947701; doi:10.1038/bjc.2016.185)
Supplement: Supplementary Table 3 [file bjc2016185x3.docx]

Supplemental Table 3. Survival from start of second-line (2L) treatment (OS2)

| Survival per 2L regimen | *nab*-P + Gem | Gem | HR (95% CI)  *P* value |
| --- | --- | --- | --- |
| Any 2L, n (%)  **Median, mo (95% CI)** | 170/421 (40)  **5.3 (4.17 to 5.78)** | 177/402 (44)  **4.5 (3.91 to 5.49)** | **1.02 (0.818 to 1.263)**  **0.886** |
| Fluoropyrimidine-containing, n (%)  **Median, mo (95% CI)** | 132/170 (78)  **5.7 (4.86 to 6.83)** | 135/177 (76)  **4.5 (3.55 to 5.49)** | **0.94 (0.730 to 1.202)**  **0.606** |
| Fluoropyrimidine combo, n (%)  **Median, mo (95% CI)** | 98/132 (74)  **6.0 (5.26 to 7.03)** | 107/135 (79)  **4.6 (3.55 to 5.88)** | **0.87 (0.657 to 1.162)**  **0.352** |
| FOLFIRINOX, n (%)  **Median, mo (95% CI)** | 18/132 (14)  **7.2 (5.75 to 9.69)** | 17/135 (13)  **3.5 (2.20 to 9.99)** | **0.67 (0.326 to 1.378)**  **0.272** |
| FOLFOX/OFF, n (%)  **Median, mo (95% CI)** | 36/132 (27)  **6.4 (4.14 to 8.84)** | 49/135 (36)  **4.5 (3.38 to 6.51)** | **0.75 (0.474 to 1.176)**  **0.206** |
| Fluoropyrimidine mono, n (%)  **Median, mo (95% CI)** | 34/132 (26)  **4.7 (3.02 to 6.83)** | 28/135 (21)  **3.9 (2.46 to 6.83)** | **1.07 (0.629 to 1.810)**  **0.808** |
| Other (than fluoropyrimidine-containing), n (%)  **Median, mo (95% CI)** | 38/170 (22)  **3.2 (2.56 to 4.93)** | 42/177 (24)  **4.8 (3.45 to 7.26)** | **1.58 (1.000 to 2.502)**  **0.047** |

FOLFIRINOX, folinic acid, 5-fluorouracil, irinotecan, and oxaliplatin; FOLFOX, folinic acid, 5-fluorouracil, and oxaliplatin; Gem, gemcitabine; HR, hazard ratio; mono, monotherapy; *nab*-P, *nab*-paclitaxel; OFF, oxaliplatin, folinic acid, and 5-fluorouracil.
