# Supplementary material for: Second-line therapy after nab-paclitaxel plus gemcitabine or after gemcitabine for patients with metastatic pancreatic cancer
Source: Br J Cancer. 2016 Jun 28;115(2):188–94. doi: 10.1038/bjc.2016.185 (PMC4947701; doi:10.1038/bjc.2016.185)
Supplement: Supplementary Table 4 [file bjc2016185x4.docx]

Supplemental Table 4. Comparison with selected key trials of second-line (2L) treatment for advanced pancreatic cancer

| **Study**  ***Patient population*** | **1L → 2L** | **n** | **Patient factors at 2L** | | **Median, mo** | | |
| --- | --- | --- | --- | --- | --- | --- | --- |
|  |  |  | **PS** | **Age, median, years** | **2L PFS** | **2L OS** | **Total OS** |
| MPACT  *Metastatic* | *nab*-P + Gem → Any | 170 | KPS 90-100, 43%^a^  KPS 70-80, 48%^a^ | 62^a^ | NA | 5.3 | 12.8 |
|  | Gem → Any | 177 | KPS 90-100, 48%^a^  KPS 70-80, 44%^a^ | 63^a^ | NA | 4.5 | 9.9 |
| PANCREOX  [Gill et al, 2014]  *Locally advanced and metastatic* | Gem → mFOLFOX6 | 54 | ECOG 0-1, 89%  ECOG 2, 11% | 65^b^ | 3.1 | 6.1 | NA |
|  | Gem → 5-FU/LV | 54 | ECOG 0-1, 93%  ECOG 2, 6% | 67^b^ | 2.9 | 9.9 | NA |
| NAPOLI-1  [Wang-Gillam et al, 2015]  *Metastatic* | Gem → MM-398 + 5-FU/LV | 117 | KPS 90-100, 59%  KPS 70-80, 39% | 63 | 3.1 | 6.1 | NA |
|  | Gem → MM-398 | 151 | KPS 90-100, 57%  KPS 70-80, 43% | 65 | 2.7 | 4.9 | NA |
| CONKO-003  [Oettle et al, 2014]  *Locally advanced and metastatic* | Gem → OFF | 76 | KPS 90-100, 54%  KPS 70-80, 46% | 62 | 2.9 | 5.9 | NA |
|  | Gem → FF | 84 | KPS 90-100, 48%  KPS 70-80, 52% | 61 | 2.0 | 3.3 | NA |

1L, first-line; 5-FU, 5-fluorouracil; FF, folinic acid and 5-FU; Gem, gemcitabine; KPS, Karnofsky performance status; LV, leucovorin; mFOLFOX6, a modified FOLFOX (folinic acid, 5-FU, and oxaliplatin) regimen; NA, not available; *nab*-P, *nab*-paclitaxel; OFF, oxaliplatin, folinic acid, and 5-FU; OS, overall survival; PFS, progression-free survival.

^a^ Data are at the end of 1L therapy.

^b^ Report does not specify whether age is given as median or mean.
